# Notes and News

**Published:** 1887-07-16

**Authors:** 


### NOTES AND NEWS.

COLLECTED AND COMMUNICATED.

At Wakefield the annual meeting of delegates in connection with the Clayton Hospital was held on Thursday, in the consulting-room of the Institution. Many of the principal inhabitants were present, and great interest was shown in the proceedings. Since the institution of the Saturday Fund in the town a grand total of £8,948 9*s*. 9*d*. has been collected, being an average of £830 a year. This amount has been subscribed exclusively by the working-classes, who may be congratulated on their energy and its successful results.

Middlesbrough-on-Tees has just held its annual demonstration on behalf of the Infirmary and the North Ormsby Cottage Hospital. Large numbers of men belonging to the various Trade, Friendly, and Temperance Societies took part in the celebration. The Mayor, who was unable to attend, sent a cheque for £5. Not long ago the Sisters at North Ormsby made an appeal for garden-seats. Have any of the iron dukes or princes of Middlesbrough sent them a dozen?

At West Ham an athletic festival has been held on behalf of the fund for the proposed local hospital. The event has proved a striking success. We can hardly imagine any more satisfactory way of rousing enthusiasm on behalf of a charitable object than that of bringing all the young people together for healthy competition in all kinds of sports. There is a great lack of healthy amusements among the people at the East-end.

On Thursday afternoon her Royal Highness the Duchess of Albany opened three new wards in the National Hospital for the Paralysed and Epileptic, constructed as a memorial to the late Duke of Albany, in Queen Square, Bloomsbury. Her Royal Highness was received by the chairman, Colonel G. C. Porter, a deputation of the medical staff, the secretary and general director, Mr. B. B. Rawlings, Colonel Duncan, M.P., the chaplain (the Rev. Mr. Moran), and others. After a short religious service by the Rev. Canon Nisbet, the Duchess was presented with an address from the board of management, who thanked her for having graced the ceremony with her presence. The Duchess declared the wards open, and then received purses in aid of the funds of the hospital. In the afternoon and evening a flower-show and concert were held, and brought together a large number of ladies and gentlemen interested in the prosperity of the hospital.

During the recent Jubilee celebrations from five to six hundred casualties were treated under the superintendence of Dr. A. O. Mackellar, chief surgeon of the metropolitan police. Fortunately few of the cases were of a serious character : more than three hundred were cases of fainting, and over twenty of sunstroke. There were several fractures of the leg, arm, and collar-bone. one dislocation of the shoulder-joint, and several wounds on the hands. Among the more serious casualties were kicks from horses, crushing of the chest, concussion of the brain, and fracture of the skull.

At Lincoln a bazaar on a scale of unusual magnificence was opened on Thursday in aid of the County Hospital. Mrs. Sibthorp, the wife of the treasurer of the institution, performed the ceremony. The west wing of the building was set apart for the purpose of the bazaar, which consists of six large stalls, a childdren's stall, a Hospital Saturday and refreshment stalls. The band of the 1st Volunteer Battalion Lincolnshire Regiment was in attendance each day, and played selections of music. The railway companies issued tickets to Lincoln at reduced rates, and every facility was given for making the bazaar a splendid success. We shall be glad to learn that the results justified the hopes of the promoters.

One of the monthly visitors at the Worcester Infirmary made a complaint at a late special meeting of the Governors that the servants' bedrooms were under the chapel in the tombs. At the request of the house committee, the Rev. T. G. Curtler visited the rooms. He found that they were under the chapel as stated, simply because the chapel happened to be on the second storey of that particular block of buildings, whereas the servants' bedrooms were on the first storey. He found that there were eight bedrooms, five single and three double-bedded, that they were spacious, lofty, well ventilated, and comfortable, and that no complaint of any kind had been made to the matron on the subject. It is well for monthly visitors to do their duty with a critical eye, but unfounded complaints of this kind savour of silliness, and should never be made.

THE following vacancies are announced this week, the amount of the salary being placed in each case after the name of the institution :—

**Resident Medical Officers.**

Carlisle Infirmary. House Surgeon.—£70.
Female Lock Hospital, Harrow Road. House Surgeon, M.R.C.S.—£100.
Hartlepools Hospital. House Surgeon and Secretary. Registered.—£60.

**Non-resident Medical Officers.**

Penrith Union. Medical Practitioner. Qualified.—£38.
Penrith Union. Public Vaccinator. Qualified.

**Trained Nurses.**

Bradford Infirmary. One.—£25.
City of London Hospital for Diseases of the Chest. Day Nurse.—£22.
Exeter Lying-in Charity. Certificated Midwife.
Hartlepool Union Workhouse. One.—£25.
Stafford Union Workhouse. One.—£25.
St. Saviour's Infirmary, S.E. Several.—£20.
Ventnor Consumption Hospital. Head.

**Probationers.**

Denbigh Infirmary. One.—£18.
Ventnor Consumption Hospital. Several.

---

THE Glasgow Medical Charities Committee are still at work. Recently they met in conference with the Committee of the Charity Organisation Society. The joint meeting decided to hold a conference with the members of the boards of management of their respective institutions and of the parochial boards, both for purposes of discussion and for the appointment of a final committee of representatives of all the bodies interested in the subject. We shall look forward with great interest to the proceedings of this committee.

---

THE managers of the Bristol Royal Infirmary are about to appoint an obstetric physician to that institution. It is evident that, notwithstanding the general progress of the times, there is a great deal of hesitancy on the part of institutions in making necessary changes. The Bristol Infirmary, and every other hospital of anything like the same magnitude, should have had an obstetric physician years ago.

---

AT the North Infirmary, Cork, a little boy named Twohig, aged three years, was brought in recently by his parents, who believed he was dead from drowning, he having been immersed in a large tub of water for a considerable time. Although the little fellow was quite unconscious when admitted, after some hours of patient labour he was brought round, and was able to be taken home in the evening. It is probable that in many cases of supposed death from drowning life is still present, and if artificial respiration were persevered with for a sufficient length of time many such cases would be restored.

---

BOURNEMOUTH took advantage of Jubilee Day to lay the foundation stone of its new hospital. The weather was fine and the town crowded, and the proceedings generally of the most pleasant character. In common with other towns Bournemouth observed the day as a general holiday, and the inhabitants of all sorts and conditions deserted for a time their business engagements, and entered most fully into the general rejoicings of the occasion.

A FEW weeks ago the foundation stone of the Merthyr General Hospital was laid by Sir W. J. Lewis, and the event was made the occasion of a grand demonstration in the town. The Volunteers, the clergy of all denominations, the benefit societies, and various other public bodies, took part in the movement. The hospital supplies a want which has been keenly felt by the poorer classes for many years past.

---

MRS. G. HOLM, whose name is well known in connection with benevolent deeds in the Kendal district, gave a treat to the patients and friends of the Kendal Memorial Hospital, in celebration of the Queen's Jubilee. Tea was partaken of in the garden behind the hospital in the most splendid of Queen's weather. The verandah was ornamented with festoons and other floral decorations. Among those present were the Venerable Archdeacon Cooper and Miss Cooper, the Rev. J. T. Suttie, the Hon. Dr. Green, Dr. and Mrs. Brumwell, Dr. and Mrs. Leeming, and others. A most pleasant Jubilee was celebrated in this homely fashion.

---

THE Queen's Jubilee at Southend has been celebrated by a bazaar and fancy fair, given at the public hall of that town in aid of the Victoria Hospital Fund. The patrons were Lord and Lady Brooke, Major Rasch, M.P., and Mrs. Rasch, Sir Frederick Heygate, Mr. Stuart Nicholson, commandant of Shoeburyness garrison, Mr. T. Kemble, Mr. J. Oxley Parker, and Mr. J. Tufnell. The total cost of the hospital is estimated at about £2,000, and prior to Wednesday about £800 had been raised. The fact that Lady Brooke had consented to open the bazaar invested it with additional interest; but, owing to the serious illness of Miss Algernon Lennox, her sister, Lady Brooke at the eleventh hour telegraphed her inability to attend. Mrs. Rasch, wife of the member for the division, was invited to perform the duty, and readily responded to the invitation. We hope the proceeds were all that the people of Southend could desire.

---

THE Miners' Hospital at Redruth, Cornwall, for the maintenance of which Lord Robartes seems to have been largely responsible, will not improbably be placed in the hands of trustees. Lord Robartes proposed to hand over the building and furniture on a lease of ninety-nine years, to persons selected from or nominated by the subscribers, at the rental of a shilling a year. The proposal was discussed with some warmth, and there was evidently a disposition on the part of several to leave the hospital, with all its responsibilities, under the wing of Lord Robartes. This, we think, displays a considerable degree of very un-English weakness and want of courage. Why should Lord Robartes alone be responsible for the maintenance of an institution which exists for the general good? It may be that there are local circumstances which give the miners a special claim upon Lord Robartes, and this he seems willing to allow by his grant of the building at a nominal rental, and his expressed intention of becoming a subscriber to the institution. We hope the committee will see their way to undertaking a responsibility which it cannot be for their honour to try to escape from.

Brentwood had the intention a short time ago of founding a convalescent home for children. That intention, it appears, has been abandoned. Mr. J. C. Goode, in addressing the subscribers and returning their subscriptions, says that although the project had fallen through as a Jubilee scheme, it is proposed to take a cottage, and make a commencement with a few children as early as arrangements can be made. He hopes therefore that those who have already offered assistance will be found faithful to the smaller venture.

Last year 168 patients were received at the Mary Wardell Convalescent Home at Brockley Hill, bringing up the total number admitted since the opening of the Home in 1884, to 306. Sir Joseph Fayrer, in moving a resolution at the annual meeting urging the necessity for increased help for the institution, said that he knew no charity which was more worthy of public support than this Home, tending, as it did, to check the spread of scarlet fever. There is still a debt of £250 on the building.

Prize-day at St. Thomas's Hospital Medical School is always a red-letter day with the medical students. This year it brought together a large and distinguished number of friends, including Sir H. W. Acland, the Regius Professor of Medicine at the University of Oxford, Sir Joseph and Lady Fayrer, Sir Thomas and Lady Crawford, the Master of the Society of Apothecaries, Sir F. Pollock, Sir Edwin and Lady Saunders, Sir Henry Pitman, Dr. Ord, and the Dean. Sir Henry Acland presented the prizes. The students number as many as 350, and the school now sends out more qualified medical men than any other medical school in England—a very proud position, as the worthy Dean pointed out, for St. Thomas's Medical School to occupy, and good evidence of the all-round education in medicine and surgery there given.

At the Nottingham General Hospital the Royal Jubilee was celebrated by a variety of festivities. All those patients who were in a condition to enjoy abundance of creature comforts were regaled both at dinner and tea with Jubilee fare. By the kindness of the chaplain, the Rev. A. H. Baynes, the regimental band of the Robin Hood Rifles was in attendance, and greatly delighted the patients. At five the band partook of a substantial tea, provided by Dr. Anderson. Music was again resumed after tea, and a most pleasant evening was appropriately brought to a close by the performance of the National Anthem.

At Bath the Mineral Water Hospital seems to be in a somewhat languishing condition for want of funds. Bath, like many other venerable places and institutions, is not what it was. But there is no doubt of the great value of Bath and its waters in certain cases of chronic rheumatism. The Bath Hospital is not a mere local charity, but receives poor persons from various parts of the country, and the pure air with the mineral water treatment are productive of the very best results in suitable cases. It seems that the wealthy visitors, who gladly acknowledge the benefits received during their stay at Bath, give little or nothing to the hospital. Would it not be well that the officials should take some steps to bring the merits and needs of the charity before the notice of visitors? If this were done intelligently and well, there can be little doubt that the result would be very satisfactory both to the donors, the hospital officials, and patients.

Sheffield has just completed its list of contributions received for the Hospital Saturday Fund. The total is £1,170 5*s*. 4*d*. Last year the amount was £1,206 6*s*. 1*d*., a decrease of £36 0*s*. 9*d*. In 1877 the sum of £1,637 2*s*. 9*d*. was received. There is thus a falling off of nearly £500 in the ten years. We are not aware that Sheffield has suffered more from trade depression than any other town of similar magnitude. The amount collected as compared with that of some other towns is highly discreditable to the metropolis of the steel trade. The working men and other inhabitants of Sheffield should look at Leeds, and feel ashamed of themselves.

The Rugby Provident Dispensary has just held its annual meeting, and the report discloses a very satisfactory year's work. There is a record of 1,533 visits paid to members, 1,667 seen and prescribed for, and 35 women attended in their confinements. All this has been provided for by the members themselves (aided by honorary subscribers) paying small sums in time of health. The number of members at present on the books is 822.

At the Hôtel Métropole, a deputation, consisting of the Mayor of Sunderland, Mr. Edwin Richardson, the ex-Mayor, Alderman R. Preston, the Earl of Durham, the Marquis of Londonderry, Mr. William Stobart, Mr. Nicholas Wood, M.P., Mr. Mackenzie, and the Town Clerk, Mr. F. M. Bowie, waited by appointment upon the Ecclesiastical Commissioners to urge the claims of the Sunderland Infirmary, more especially of the Hartley Memorial Wing. The Ecclesiastical Commissioners are the owners of extensive coal royalties in the neighbourhood of Sunderland. The Earl of Durham introduced the deputation, and set forth the present position of the infirmary, explaining what was proposed to be done by way of enlarging the institution. The Marquis of Londonderry, and other members of the deputation, followed with observations as to the excellent management of the infirmary. It was said that the Commissioners had offered to contribute the sum of £250 towards the extension, but the deputation strongly urged upon them that, considering the large revenue they derived from the immediate neighbourhood, and the special needs of the case, that sum should be largely increased. Memorials signed by upwards of 6,500 of the miners were presented to the Commissioners in support of the object of the deputation. Lord Stanhope, as Chairman of the Commissioners, promised that the subject should receive their earnest consideration. The Ecclesiastical Commissioners are the custodians of a vast amount of wealth, and might very well afford to increase their proposed contribution fourfold.

